# Septic Shock Due to Candidemia: Outcomes and Predictors of Shock Development

**DOI:** 10.4021/jocmr536w

**Published:** 2011-04-04

**Authors:** Jorge A Guzman, Ronny Tchokonte, Jack D Sobel

**Affiliations:** a Section of Critical Care, Respiratory Institute, Cleveland Clinic, Cleveland, USA; b Pulmonary and Critical Care, Wayne State University, Detroit, USA; c Division of Infectious Diseases, Wayne State University, Detroit, USA

## Abstract

**Background:**

The present report describes the outcomes of a cohort of patients with *Candida* induced septic shock.

**Methods:**

Retrospective analysis of individuals who had at least one positive blood culture for *Candida* species ≥ 48 h after ICU admission. Data from patients that developed septic shock within 48 hr of the positive blood culture were compared to non-shock candidemic patients. Patients with a concomitant bacteremia and/or endocarditis were excluded.

**Results:**

Fifteen patients with *Candida* induced septic shock were studied and compared to 35 candidemic patients without shock. Overall mortality was 76% (87 % among those who had shock). A high proportion of non-*albicans Candida* species causing fungemia (74%) was observed. All patients with shock were receiving antibiotics but not antifungal treatment at the time of shock development, eight were on parenteral nutrition, six on steroids and nine had a cancer history. High dose fluconazole was the most common initial treatment provided. Four patients died before receiving any antifungal treatment. Time in ICU before the development of candidemia was identified as a predictor of shock development (higher chance if fungemia developed < 7 days after ICU admission).

**Conclusions:**

Septic shock due to invasive candidiasis is a near fatal condition. No conventional risk factors were identified to predict shock development other than time (shorter) spent in ICU before the development of candidemia. We encourage clinicians to consider the initiation of appropriate empiric antifungal treatment in high-risk patients who develop septic shock while on antimicrobial treatment.

**Keywords:**

Septic shock; Candidemia; Outcome; Predictor

## Introduction

*Candida* is an increasing cause of bloodstream infection, causing significant mortality and morbidity. Its overall incidence rose fivefold in the past ten years and remains the fourth leading cause of nosocomial bloodstream infection in the United States, accounting for 8% of all bloodstream infections acquired in hospitals [1-3]. Furthermore, a change in the epidemiology has also been noted. Species other than *C albicans* have increased and surpassed *C albicans* in incidence in some tertiary care centers [4-7]. Some of these emerging species have been correlated with increased virulence [8], and in some reports with increased mortality [9]. Despite the availability of effective antifungal therapy, crude mortality in the last decade has remained high, ranging from 36 to 90% [9-11].

A number of predisposing factors have been associated with development of invasive candidiasis and clinical predictors of poor outcome have been reported [12-14]. Nevertheless, studies describing the clinical course of patients developing candidemia are scant [15], and limited data exist of the outcomes of patients who develop septic shock due to invasive candidiasis [16]. In general, when patients develop septic shock, bacterial causes are exclusively considered and no effort to empirically treat *Candida* species is made.

The present report describes a cohort of patients with *Candida* induced septic shock. In order to identify predictors of shock development, data obtained from patients in shock were compared to a group of patients who had ICU acquired candidemia without shock.

## Materials and Methods

The Human Investigation Committee (HIC) of Wayne State University approved the study protocol. The medical records of patients who had at least one positive blood culture specimen of *Candida* spp. collected after their ICU admission at Harper University Hospital over a five year span (1998 - 2003) were reviewed. Case patients were defined as individuals who had at least one positive blood culture for *Candida* species grown ≥ 48 h after ICU admission and in whom septic shock developed within 48 hrs of a positive blood culture. Patients who had a blood culture positive for a concomitant bacterial pathogen (i.e., mixed infection) and patients with endocarditis were excluded.

### Definitions

Septic shock: acute circulatory failure characterized by persistent arterial hypotension (systolic arterial pressure below 90 mm Hg, a MAP < 60 mm Hg, or a reduction in systolic blood pressure of > 40 mm Hg from baseline, despite adequate volume resuscitation, or the need for vasopressors in the absence of other causes for hypotension) in the presence of candidemia [17]. ICU acquired candidemia: Positive blood culture for *Candida* spp. grown ≥ 48 hrs after ICU admission. Colonization was defined as presence of *Candida* spp. in a non-sterile site. Candiduria as the presence of ≥ 10^4^ cfu/mL of *Candida* spp. in urine. Airway colonization: *Candida* spp. isolated from tracheal aspirates or bronchoalveolar lavage specimens. Catheter-associated candidemia was determined as ≥15 cfu of *Candida* spp. isolated from a central venous catheter tip.

### Study variables

Study population characteristics and data extracted included demographics, age, and sex of the patients, reason for ICU admission, APACHE (Acute Physiology and Chronic Health Evaluation) II score at admission, length of stay (LOS) in ICU, hospital LOS, LOS prior to ICU admission, time in ICU before development of candidemia, predisposing risk factors for candidemia such as diabetes mellitus, broad spectrum antibiotic treatment (for > 3 days) during the hospital stay, chronic renal failure, liver disease, *Candida* colonization, systematic administration of glucocorticosteroids for any reason in the last 30 days before ICU admission (regardless of dose), total parenteral nutrition administration, central venous catheter (CVC) placement (for > 2 days), the day of candidemia occurrence, and outcome (i.e., dead or alive at ICU discharge).

### Statistical analysis

The [chi]^2^ test or Fisher’s exact test was used to evaluate categorical variables and the Student’s *t*-test to evaluate continuous variables. Multiple logistic regression analysis was performed to identify independent variables associated with development of shock. Potential risk factors were included in the analysis if they were associated with the dependent variables in the univariate analyses at a statistical level P < 0.2. A P < 0.05 was considered statistically significant in the multivariable models to show association between various potential risk factors and the dependent variable. An empirical receiver operating curve (ROC) was created for each predictor. Also, sensitivity and specificity for each predictor were calculated. The optimal threshold was chosen in such a way that the threshold made the resulting binary prediction as close to the perfect operating point as possible. The Euclidean method was used to measure the distance between an observed point on the ROC curve and the perfect point.

## Results

**Figure 1.**
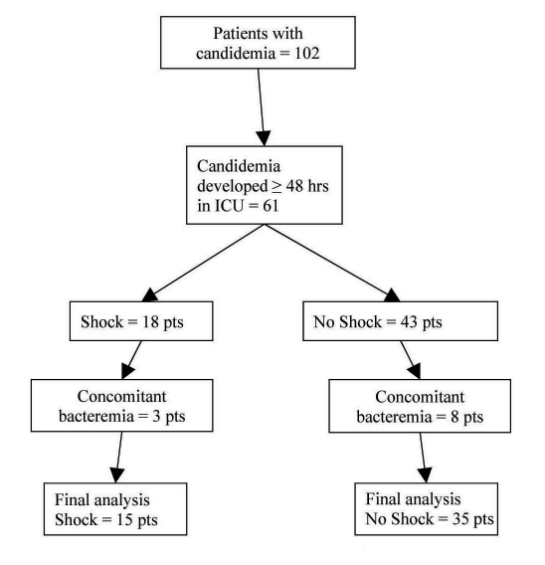
Flowchart of study subjects.

Fifteen patients with *Candida* induced septic shock were identified and included in the study. Data obtained from this group was then compared to thirty-five patients with ICU acquired candidemia who did not develop shock (Fig. 1). The main characteristics of patients who developed shock are shown in Table 1. Patients with shock had higher illness acuity, most had a history of cancer (60%) and four were neutropenic at the time of ICU admission. Candidemia in patients with shock developed 5.5 ± 3.8 days after ICU admission; only three patients had a positive blood culture within 48 hrs of ICU admission. Mortality among patients with *Candida* induced septic shock was 87%. All patients were receiving antibiotic treatment (mean number of antibiotic agents 4.1 ± 1.7) for 4.2 ± 3.9 days prior to the development of candidemia. Six (40%) patients were on corticosteroids (mean duration of treatment prior to candidemia 4.9 ± 2.6 days) and eight (53%) were on parenteral alimentation at the time of development of candidemia. All patients had at least one CVC in place. Ten patients were initially treated with high dose fluconazole (≥ 12 mg/kg/day), one with amphotericin B, and four patients died before they received any antifungal treatment (culture results positive post mortem). Eight of the eleven treated patients cleared the fungemia after 7.4 ± 7.9 days of antifungal treatment, and 3 patients had persistent candidemia. Antifungal therapy was subsequently changed in 6 patients (five placed on deoxycholate amphotericin B and 1 on voriconazole).

**Table 1:** Patients With Candidemia Associated Septic Shock: Characteristics and Outcome

Age	Sex	ICU admission diagnosis	*Candida* spp.	Predisposing factors for candidiasis					**Outcome**
				Cancer	PN	CRF	DM	Liver Ds	
50	Male	Septic shock	*Albicans*	Yes	---	---	---	Yes	Deceased
71	Female	Post cecal mass resection	*Albicans*	Yes	Yes	---	---	---	Deceased
84	Male	Post exploratory laparotomy for colonic perforation	*Albicans*	---	Yes	Yes	Yes	---	Deceased
83	Female	Septic shock	*Albicans*	---	---	---	---	---	Deceased
59	Male	Gastrointestinal bleeding (admitted to the hospital with urosepsis)	*Albicans*	---	---	---	Yes	Yes	Deceased
63	Male	Aspiration pneumonia	*Glabrata*	---	---	Yes	---	---	D/C LTACH
54	Female	Post clipping of cerebral aneurysm	*Glabrata*	---	Yes	---	---	---	D/C Rehab
77	Male	Respiratory failure-pneumonia	*Glabrata*	---	Yes	---	---	---	Deceased
74	Male	Gastrointestinal bleeding	*Glabrata*	Yes	Yes	---	---	---	Deceased
50	Female	Endobronchial radiation treatment	*Glabrata*	Yes	---	---	---	---	Deceased
56	Female	Post exploratory laparotomy (colon and stomach resection)	*Glabrata*	Yes	---	---	---	---	Deceased
40	Female	Sepsis - neutropenia	*Lusitaniae*	Yes	---	---	---	---	Deceased
64	Male	Septic shock	*Albicans*	Yes	Yes	---	---	---	Deceased
57	Male	Respiratory failure post peripheral stem cell transplant	*Glabrata*	Yes	Yes	---	---	---	Deceased
51	Female	Neutropenic fever and septic shock	*Glabrata*	Yes	Yes	---	---	---	Deceased

CRF: chronic renal failure; DM: diabetes mellitus; PN: parenteral nutrition; Liver Ds: liver disease; D/C: discharged; LTACH: long term acute care hospital

Table 2 shows data for the whole cohort and compares candidemic patients with and without shock. Overall mortality was very high (76%). Patients in shock had a higher illness acuity and higher mortality. ICU stay prior to the development of candidemia was shorter in patients who developed shock and so was their overall hospital LOS (presumably because these patients died more rapidly). Duration of candidemia was identical in both groups. Although there were proportionally more patients with *Candida* albicans who developed shock, no significant statistical difference in species was noted between the two groups. Four patients in the shock group (27%) had documented *Candida* colonization (three patients had combined urine and sputum, one patient had only sputum colonization), whereas eighteen patients (51%) in the no-shock group were colonized (eight patients urine, 10 sputum and 5 patients catheter-related). The difference did not reach statistical significance (P = 0.135).

**Table 2:** Patient Age, Acuity, Outcomes and Lengths of Stay

	All Candidemias (n = 50)	No shock (n = 35)	Shock (n = 15)	P Value
Age (years)	61 ± 14	60 ± 14	62 ± 13	0.64
Apache II	22.6 ± 8.3	21.1 ± 7.2	26.5 ± 9.7	0.04
Mortality (%)	76	71	87	0.30
Hospital LOS (days)	35.6 ± 19.8	40.1 ± 19.1	25.1 ± 17.9	0.01
ICU LOS (days)	22.0 ± 16.0	24.0 ± 16.5	17.4 ± 14.3	0.18
Hospital LOS prior to ICU (days)	7.9 ± 10.5	9.3 ± 11.5	4.4 ± 6.7	0.13
Time in ICU prior to candidemia	13.5 ± 13.2	16.9 ± 14.4	5.5 ± 3.8	0.004
Documented colonization (patients)	22 (44%)	18 (51%)	4 (27%)	0.13
*Candida* spp. patients (%)				
*Albicans*	14 (26%)	8 (23%)	6 (40%)	0.30
Non-*albicans*	36 (74%)	27 (77%)	9 (60%)	
Time to clearance (days)	8.6 ± 8.3	7.4 ± 7.9	9.0 ± 8.6	0.64

LOS: Length of stay

**Table 3:** Multiple Logistic Regression Analysis

	Odds ratio	95% CI	P Value
APACHE II score	1.007	0.882 - 1.149	0.919
Hospital LOS	0.902	0.805 - 1.010	0.075
ICU LOS	1.119	0.988 - 1.267	0.076
Hospital LOS before ICU	0.965	0.819 - 1.138	0.675
ICU LOS prior to candidemia	0.714	0.531 - 0.960	0.026
*Candida* colonization	0.291	0.037 - 2.258	0.237

Hosmer-Lemeshow statistic: 3.175 (P = 0.923); Likelihood ratio test statistic: 28.474 (P ≤ 0.001) ICU: Intensive care unit; APACHE: Acute Physiology and Chronic Health Evaluation; LOS: Length of stay

**Figure 2.**
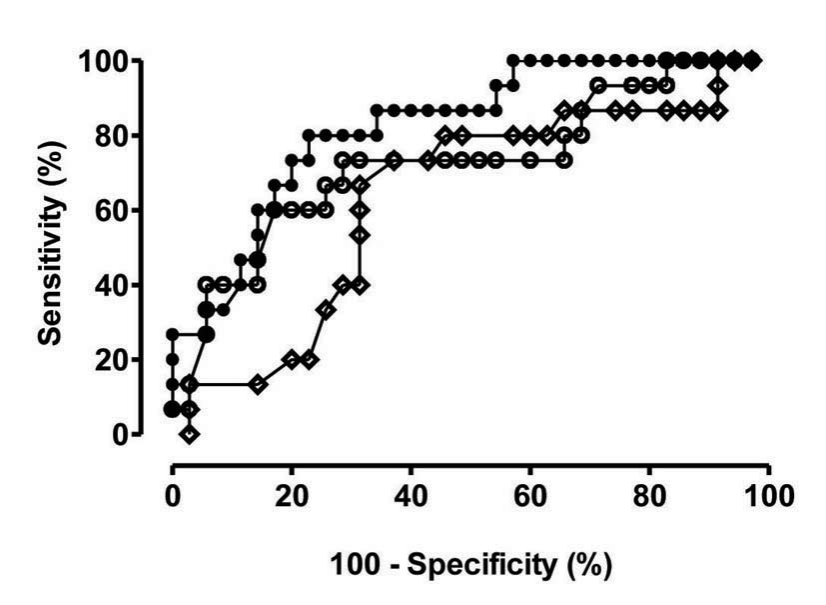
Receiver operator curves for the three predictors with statistical significance. Closed circles: ROC for time in ICU prior to candidemia; Open circles: ROC for ICU length of stay (LOS); Open diamonds: ROC for hospital LOS

Table 3 shows odd ratios and confidence intervals for shock development for the parameters identified by univariate analysis. Time in ICU before the development of candidemia reached significance as predictor of shock development. Area under the receiver operating curve was 0.826 (Fig. 2). The threshold of < 7.2 days in ICU before the development of candidemia allowed a discrimination between shock development and no shock with a sensitivity of 73.3% and a specificity of 80.0%. A trend was also noted for hospital and ICU LOS prior to development of candidemia, the lack of statistical significance may be related to the limited sample size. Judging by the values of the Hosmer-Lemeshow and the likelihood ratio statistic, the model appears well calibrated.

## Discussion

The present study shows that *Candida* induced septic shock is a nearly fatal condition with mortality rate that triples that of bacterial septic shock. Estimation of the true incidence of *Candida* induced septic shock is not possible from this study, although the percentage of patients with shock i.e. 30% in our ICU cohort is similar to the 27% and 23% frequency previously reported [15, 18]. The comparable rates strengthen the selection criteria employed to identify case patients in this retrospective cohort and highlight the high risk of progression to septic shock among critically ill patients who develop candidemia, a fact ignored by many clinicians.

The high mortality observed among ICU patients with candidemia has been attributed to a number of factors, including APACHE scores, inadequate initial fluconazole dosing, delayed administration of antifungal therapy due to slow fungal growth or insensitive culture methods, or retention of central venous catheters [12, 18]. High dose fluconazole was the initial treatment in all but one patient. Although current treatment guidelines suggest use of echinocandin agents or polyenes in hemodynamically unstable patient [19], these recommendations were published after the study occurred (1988 - 2003) and before the availability of echinocandins. It remains speculative whether the outcomes observed in this cohort have any relationship to the treatment provided. Nevertheless, it is still feasible that the high mortality observed was a consequence of using a fungistatic agent such as fluconazole rather than fungicidal echinocandin or polyene agents. A more likely explanation is the time that our patients were without antifungal treatment awaiting blood cultures to turn positive. This emphasizes the need to introduce sensitive diagnostic assays that facilitate earlier pathogen identification. Supporting this concept is the fact that almost a third of our patients died before isolating the organism responsible for septic shock and therefore did not receive antifungal treatment. All patients were being treated with multiple antibiotic agents at the time of shock development, begging the question whether clinicians should consider empiric antifungal therapy in patients who develop shock while receiving broad spectrum antimicrobial agents.

Although a number of studies have described risk factors for development of candidemia, none have specifically identified factors predisposing to development of shock [13, 14, 20, 21]. Using a multiple regression analysis we were able to identify the time spent in ICU until the development of candidemia as the only parameter that reached statistical significance as shock predictor. ROC analysis suggests using a threshold of 7 days in the unit as the best way to identify who may develop shock due to candidemia. While this may be an important first step alerting clinicians that there may be a higher chance for shock development in patients who develop candidemia early on the course of ICU stay, we realize that the limited sample size precludes developing a more powerful model for better prediction of shock.

While not statistically significant, proportionally more patients with *C albicans* developed shock. This observation was previously reported by Hadley et al, where 7 out of 10 described episodes of candidemia and shock were caused by *C. albicans* [16]. Although it is tempting to postulate that inherent properties of each species would induce varying degrees of inflammatory response and thus explain the higher incidence of shock observed by *C. albicans*, this hypothesis was not supported by Wisplinghoff et al [15]. The authors reported similar initial and most severe inflammatory responses with both *C albicans* and non-*albicans* Candida spp. over time. The limited sample size precludes reaching any definitive conclusion, although these data may be hypothesis generating and lead to further investigation to determine whether different species trigger different host response. Although there has been a reported increase in the incidence of non-albicans *Candida* fungemia [6, 10], the observed ratio of non-*albicans* to *C. albicans* in our cohort is extremely high. It remains unknown whether this is a reflection of antibiotic and antifungal use within our medical center, or a reflection of severity of disease in patients acquiring candidemia. Nonetheless, in selecting the initial early empiric antifungals one should recognize the high frequency of non-*albicans Candida* species with intrinsic reduced fluconazole susceptibility.

Our study has limitations. Because of its retrospective nature, the definition of shock induced by candidemia may be disputed, although we only included patients who had hypotension not explained by any other reason than infection and the percentage of patients progressing to shock is similar to previously described [15]. Similarly, colonization was not documented in all patients as it is not routine to do surveillance cultures for *Candida* in the US. The study period preceded the introduction of echinocandins into clinical use. In spite of these limitations, this is to our knowledge one of the largest cohorts of patients with *Candida* induced septic shock and the first that has attempted to identify predictors of shock development thus far.

In summary, septic shock due to candidemia in the ICU setting is associated with extremely high mortality. No conventional risk factors were identified to predict shock development other than the time spent in ICU until the development of candidemia. Given the high mortality associated with candidemic septic shock, consideration should be given for initiating empiric broad spectrum cidal antifungal therapy in high risk ICU patients already receiving and not responding to antibiotic therapy. This therapeutic approach should be validated by prospective studies.
